# Diagnosis of depression among adolescents – a clinical validation study of key questions and questionnaire

**DOI:** 10.1186/1471-2296-8-41

**Published:** 2007-07-13

**Authors:** Ole R Haavet, Kaj S Christensen, Manjit K Sirpal, Wenche Haugen

**Affiliations:** 1 Institute of General Practice and Community Medicine University of Oslo, Pb. 1130 – Blindern, N-0318 Oslo, Norway; 2 Research Unit for General Practice, Vennelyst Boulevard 6, DK-8000 Århus C, Denmark

## Abstract

**Background:**

The objective of the study is to improve general practitioners' diagnoses of adolescent depression. Major depression is ranked fourth in the worldwide disability impact.

**Method/Design:**

Validation of 1) three key questions, 2) SCL-dep6, 3) SCL-10, 4) 9 other SCL questions and 5) WHO-5 in a clinical study among adolescents. The Composite International Diagnostic Interview (CIDI) is to be used as the gold standard interview. The project is a GP multicenter study to be conducted in both Norway and Denmark. Inclusion criteria are age (14–16) and fluency in the Norwegian and Danish language. A number of GPs will be recruited from both countries and at least 162 adolescents will be enrolled in the study from the patient lists of the GPs in each country, giving a total of at least 323 adolescent participants.

**Discussion:**

The proportion of adolescents suffering from depressive disorders also seems to be increasing worldwide. Early interventions are known to reduce this illness. The earlier depression can be identified in adolescents, the greater the advantage. Therefore, we hope to find a suitable questionnaire that could be recommended for GPs.

## Background

The proportion of adolescents suffering from depressive disorders seems to be increasing worldwide [1]. A standardised scale assessing the extent of disability caused by all major medical disorders ranked major depression fourth in worldwide disability impact [2]. Thus an improvement of GPs' ability to recognize depression is necessary to improve mental health care among adolescents.

Because such a large proportion of teenagers is affected by depression and because interventions are known to reduce this illness [3,4], the earlier depression can be identified in adolescents, the greater the advantage. GP's screening of adolescents is not systematized in the same way as it has been for younger age groups in, for example, Denmark. One goal of the project is to increase awareness of this forgotten group of patients. Another purpose is to provide information that will increase the probability of making the correct diagnosis.

Although the majority of adolescents in Nordic countries enjoy good health, a large number experience depression [5], a condition that is correlated with physical illness [6]. There is reason to believe, in fact, that up to 18% of the age group 14–16 years experiences symptoms of depression [7-9]. A French study has shown that 30% of teenagers has at least one depressive symptom and that only 35% of teenagers with major depression seeks professional treatment [10]. The literature indicates that the prevalence of major depression varies from 0,4 to 8,3% [11], with a boy/girl ratio of 1/2,5 [12,8.9]. Depression in teenagers is associated with decreased levels of functioning in the home environment, socially, and at school; higher levels of drug abuse; and increased risk of illness and early death in adult life [13].

Kramer et al. [14] has shown that only one in five adolescents with depressive disorder consulting a GP is correctly diagnosed (sensitivity 20%), probably because neither the adolescent nor their parents can comprehend or explain the psychological problems. This situation suggests that general practitioners require reliable instruments for the diagnosis of depression [15].

Ethnic factors seem to affect clinical work, due to such differences as language, religion, nationality, cultural values, and understanding of health/illness. Hinton et al. [16] illustrate asylum seekers' special use of the "idiom", Wind Overload, to describe the background of their psychological illness. Bhui et al. [17] have shown that the Amritsar Depression Inventory, a questionnaire developed and validated in Punjab, was no better at finding depression among Punjabi adults living in England than was the 12-item General Health Questionnaire (GHQ). Perhaps ethnicity is not a significant variable in the choice of a diagnostic instrument, or perhaps the sample chosen for the study was well integrated into English culture.

Subscales from the Symptom Check List, Scl-dep6 and Scl-10 are instruments that have been used in epidemiological studies. SCL-dep6 has been validated for general practitioners in a Danish study [15], for instance. SCL-10 has been used since 1992 in an adolescent study at NOVA (Norwegian Institution for Research of Growth, Health and Ageing); as well as in the Youth-Hubro Study 2000/2001 and 2004 (Institute of General Practice and Community Medicine, University of Oslo). The latter study included all students from the 10th year classes in Oslo in both 2000 and 2001, and was repeated with the same student group in 2004. SCL-dep6 and SCL-10 are both candidates for use in general practice. The WHO Wellbeing Index, WHO-5, is widely used in epidemiological studies and has been validated and recommended for depression screening in primary care [18].

Three key questions have been shown to improve the diagnosis of depression in adults in a study published in BMJ [19] in which GP sensitivity increased to 79% and specificity to 94%. In our study we examine the possibility that this finding may also be true for the adolescent group.

A two-step selective screening procedure is shown to be effective in diagnosing depression according to the literature review by the Cochrane library [20]. The first step is a validated questionnaire. Patients who score above a chosen value on that instrument are followed up with an interview by, for example, a GP.

Given the studies summarized here, we attempt to validate 1) the three key questions, 2) SCL-dep6 and 3) SCL-10, 4) 9 other SCL questions and 5) WHO-5 [18], in a clinical study among adolescents. The study also examines the effects of gender and ethnic background on validation, adolescents' preference for web-based versus paper questionnaires, and the success of our screening procedure in improving GPs' recognition of depression among adolescents. Finally, we hope to find a suitable questionnaire that could be recommended for implementation in the electronic medical records of GPs.

## Methods/Design

The project is a collaboration between the Research Unit for General Practice, University of Aarhus and the Institute of General Practice and Community Medicine, University of Oslo.

The three key questions were published in BMJ [19] and the questionnaires SCL-dep6 and SCL-10 are short versions from SCL-90. The two questionnaires had already been validated against a number of recognised questionnaires such as SCL-25 [21] and SF-36. SF-36 is used in Eurobarometer in 17 EU countries, of which Denmark is one. The three questions from Arroll et al. are; "During the past month have you often been bothered by feeling down, depressed or hopeless?" and "During the past month have you often been bothered by little interest or pleasure in doing things?" and "Is this something with which you would like help?" The first two questions have two possible responses: "yes" or "no". The last question allows for three possible responses: "no";" yes, but not today"; or "yes". We added nine more questions from SCL-90 and five questions from WHO-5 as supplements to the key questions and questionnaires.

The Composite International Diagnostic Interview (CIDI), a well known clinical instrument for measuring depression based upon DSM-IV and ICD-10 is to be used as the gold standard interview [22]. The interviewers will be certified as CIDI interviewers before the study is conducted.

**Figure 1:**
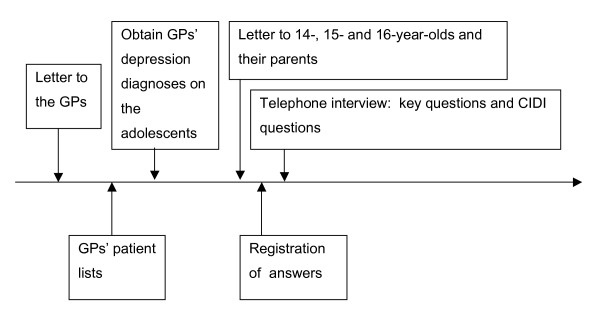
Flow diagram.

The project is a GP multicenter study to be conducted in both Norway and Denmark. Inclusion criteria are age (14–16) and fluency in the Norwegian and Danish language. A number of GPs will be recruited from both countries and at least 162 adolescents will be enrolled in the study from the patient lists of the GPs in each country, giving a total of at least 323 adolescent participants (Figure 1).

Every participating GP in Norway will be asked to make a list with three columns: 1) the name and birth number for each participant, 2) the GP's assessment of whether or not the participant is recognized as being depressed and 3) participant number (code for identification and code for login on the web questionnaire). The patient's GP will have the complete list, and the research group will use the last two parts of the list.

In Denmark the study group will provide every adolescent a code. The GP receives a list with the names of the adolescents and will be asked to mark the patients who have experienced depression during the previous month.

A standard letter of invitation to participate will be sent to the adolescent age group 14, 15, 16; in Norway a letter will also be sent to the parents of adolescents 14 and 15 years of age. The logo of the University in Oslo and the Research Unit for General Practice in Aarhus will be used on the letterhead. The letter will contain standardised information to the adolescents and their parents, a questionnaire and a pre-stamped envelope. An agreement form to be signed by parents and returned to the GP will also be enclosed with the letter to the 14- and 15-year-olds in Norway.

The invitation letter contains information about the intention of the study and its procedure. In Denmark, the adolescents' participation can be interpreted as agreement to participate in the study. In Norway, the 14- and 15-year-olds and their parents must sign the agreement form to indicate their participation, which will then be returned to the GP.

The adolescent can respond by using the forms sent by post or by using the web site on the Internet [23]. The response will be recognised by the login code and participation number. In cases in which major depression requiring immediate treatment is found, the study group will contact the GP, who can then correlate the participation number with the patient's data.

The adolescents in this study will provide in their questionnaire the telephone number by which they wish to be contacted, and a member of the study group will call the adolescent when the questionnaires have been received. The time span between the first letter/agreement form and the telephone interview will be noted. The telephone interview will begin with the three key questions, followed by the CIDI interview. From the CIDI interview the electronic version of the depression module will be used. The results will be registered electronically.

The data will be collected in a data bank directly from the web site. All the data collected in CIDI interviews and the paper versions of the questionnaire will be fed into the same data bank.

Both external and internal validity will be examined – internal validity with the use of Chronbach's alpha and the Mokken analysis, and external validity through Receiver Operating Curves (ROC), with specifying measurement for sensitivity, specificity and predictive values. The diagnostic precision of the key questions (sensitivity, specificity) will be calculated in the same manner. The GPs diagnostic precision will also be compared with the questionnaire and the key questions.

The approximate number of adolescents needed for the study was calculated using the 2 × 2 table, 0,05 probability ratio and *p *= approximated sensitivity with the formula n = *p*(1 - *p*)/(0,05/1,96)2. With *p *= 70%, the n value is 323.

### Ethics and study approval

The study was approved by The Danish Data Protection Agency and The Ethical Committee in Denmark, and The Norwegian Social Science Services and The National Committees for Research Ethics in Norway. The Danish ethics committee stated that no approval was needed, while the Norwegian ethics committee only required consent from the participants and parents of the children 14 and 15 years old. The project protocol and articles with results will be submitted for publication in refereed medical papers or databases. If a response from an adolescent taking part in the study should indicate risk for major depression, the GP concerned will be contacted, and will be able to identify the person by participation number and provide the necessary treatment.

### Presentations

Preliminary results will be presented at a symposium on the 15th Nordic Congress of General Practice, Reykjavik, Iceland 13–16 June, 2007.

### Articles planed

The following articles are planned (First author is indicated by the initials).

Diagnosis of depression among adolescents with the aid of SCL-dep6 (KSC)

Diagnosis of depression among adolescents with the aid of SCL-10 (ORH)

Diagnosis of Depression among adolescents with the aid of three key questions (WH)

Diagnosis of Depression among adolescents – Is ethnicity of importance? (MKS)

Psychiatric standard questionnaire, paper and web-based: Which do adolescents prefer? (WH)

Does screening improve diagnosis of adolescents' depression among in general practice? (MKS)

This protocol is written in Norwegian, Danish and English, and the English version is to be sent to BioMed Central database with the intention of publication. (ORH)

## Competing interests

The author(s) declare that they have no competing interests.

## Authors' contributions

ORH had the original idea for the study and made the first draft of the study protocol in cooperation with KSC. All the authors have participated in the planning of the study, in preparing the manuscript, and all the authors have approved the final version.

## Pre-publication history

The pre-publication history for this paper can be accessed here:


